# Cucurbitacin D exhibits potent anti-cancer activity in cervical cancer

**DOI:** 10.1038/srep36594

**Published:** 2016-11-08

**Authors:** Mohammed Sikander, Bilal Bin Hafeez, Shabnam Malik, Abdulrhman Alsayari, Fathi T. Halaweish, Murali M. Yallapu, Subhash C. Chauhan, Meena Jaggi

**Affiliations:** 1 University of Tennessee Health Science Centre, Memphis, TN, 38163, USA; 2 King Khaled University, College of Pharmacy, Box 188, Abha, 61441, Saudi Arabia; 3 South Dakota State University, Brookings, SD, 57007, USA

## Abstract

In this study, we for the first time, investigated the potential anti-cancer effects of a novel analogue of cucurbitacin (Cucurbitacin D) against cervical cancer *in vitro* and *in vivo.* Cucurbitacin D inhibited viability and growth of cervical cancer cells (CaSki and SiHa) in a dose-dependent manner. IC50 of Cucurbitacin D was recorded at 400 nM and 250 nM in CaSki and SiHa cells, respectively. Induction of apoptosis was observed in Cucurbitacin D treated cervical cancer cells as measured by enhanced Annexin V staining and cleavage in PARP protein. Cucurbitacin D treatment of cervical cancer cells arrested the cell cycle in G1/S phase, inhibited constitutive expression of E6, Cyclin D1, CDK4, pRb, and Rb and induced the protein levels of p21 and p27. Cucurbitacin D also inhibited phosphorylation of STAT3 at Ser727 and Tyr705 residues as well as its downstream target genes c-Myc, and MMP9. Cucurbitacin D enhanced the expression of tumor suppressor microRNAs (miR-145, miRNA-143, and miRNA34a) in cervical cancer cells. Cucurbitacin D treatment (1 mg/kg body weight) effectively inhibited growth of cervical cancer cells derived orthotopic xenograft tumors in athymic nude mice. These results demonstrate the potential therapeutic efficacy of Cucurbitacin D against cervical cancer.

Cervical cancer is the fourth most common cause of cancer-related deaths in women worldwide. According to the American Cancer Society, 4,120 deaths will occur and 12,990 new cases of cervical cancer are expected to be diagnosed in the year of 2016 in the United States^1^. Persistent infection with high-risk human papilloma viruses (HPVs) has been recognized as risk factors for developing cervical cancer. Among all HPVs, HPV-16 and −18 are the major risk factors for developing 70% of cervical cancer in women^2^. Numerous lines of evidence suggest that PTEN/PI3K/AKT/STAT3 signaling pathways play vital role in the process of cervical carcinogenesis^3^,^4^,^5^. A recent study has shown activation of PI3K/AKT and destabilization of PTEN protein involved in cervical tumorigenesis^5^. It has been reported that STAT3 regulates PI3K/AKT signaling pathways and is involved in poor prognosis of cervical cancer^4^,^5^,^6^,^7^,^8^. Moreover, various oncogenic signaling molecules and micro RNAs (miRNAs), small noncoding RNAs that modulate the expression of oncogenic and tumor suppressive genes, also play an important role in the development of cervical carcinogenesis^9^. Thus targeting these oncogenic signaling pathways and miRNAs could be a novel approach for the treatment of cervical cancer.

At present, chemotherapy is one of the most practiced approaches for the treatment of advanced metastatic cervical cancer. However, clinical application of this approach often features serious challenges involving development of chemoresistance and toxic side effects. Thus, there is an urgent need to develop a new non-toxic modality for the prevention and treatment of cervical cancer.

Naturally occurring dietary compounds have gained increasing attention for the prevention of various type of cancers^10^,^11^,^12^,^13^ including cervical cancer^14^,^15^. Cucurbitacins are tetracyclic triterpenes commonly found in *Cucurbitaceae* family which have been used in conventional medicine for decades^16^. Although, cucurbitacins exhibit moderate to high toxicity, it is remarkable to mention that the toxic dose for Cucurbitacins is much larger than the active dose thus increasing their potential as a therapeutic agent^17^. Cucurbitacins have potential to be used as possible bioactive agents for inhibiting cancer progression and these compounds contain structural improvements for future potential chemotherapeutic modalities. Various studies have demonstrated that cucurbitacin analogues have a broad range of biological effects including anti-inflammatory, hepatoprotective, anti-cancer and antioxidant activities^18^. It has been shown that Cucurbitacin E inhibits the viability of pancreatic cancer cells and induces apoptosis *via* suppression of STAT3 phosphorylation and up-regulation of tumor suppressor p53^19^,^20^. Cucurbitacin E has also been shown to inhibit proliferation of prostate cancer cells and cause disruption of the cytoskeleton structure^21^. Cucurbitacin B is found in many Cucurbitaceous plant species and is one of the abundant forms of cucurbitacins^22^. Cucurbitacin D is one of the analogue of cucurbitacins which has shown anti-cancer activity against various types of cancer^23^,^24^,^25^,^26^,^27^. Most of the studies have revealed anti-cancer effects of Cucurbitacin D *via* induction of apoptosis and suppression of constitutive activation of NF-κB and STAT3. A study has shown chemosensitization effect of Cucurbitacin D in breast cancer cells *via* inhibition of STAT3 and NF-κB^24^. Cucurbitacin D has also been reported as a potent disruptor of the HSP90 chaperone machinery^28^. However, no study has demonstrated its anti-cancer effect against cervical cancer so far. In this study, we show for the first time, potential anti-cancer activity of Cucurbitacin D against cervical cancer *in vitro* and *in vivo* model systems.

## Results

### Cucurbitacin D inhibits proliferation and clonogenic potential of CaSki and SiHa cells

**Figure 1:**
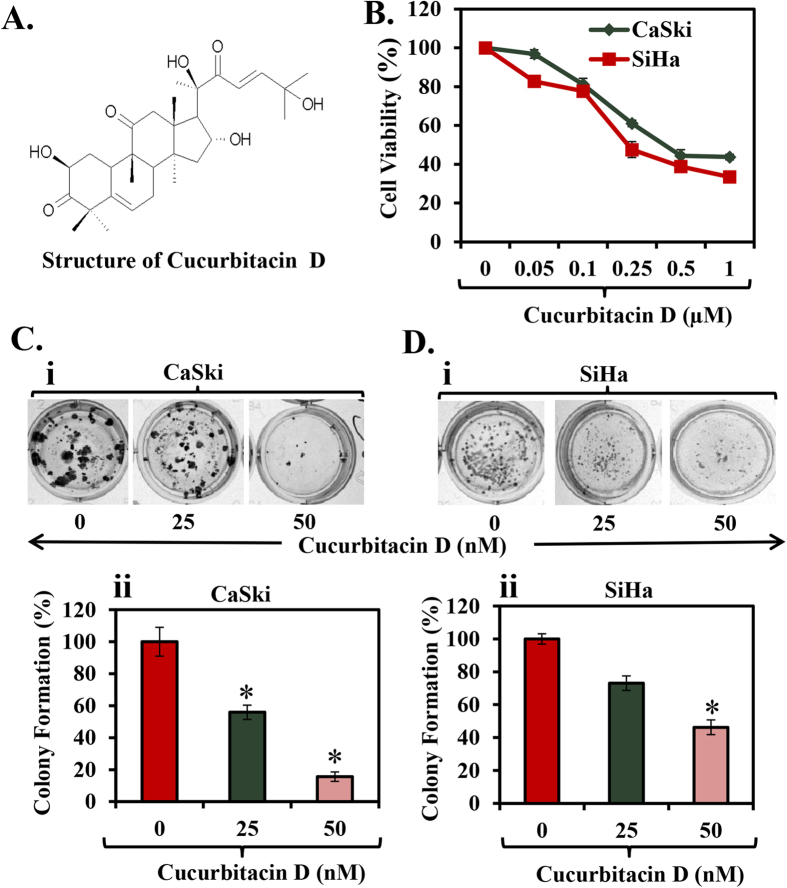
Effect of Cucurbitacin D on proliferation and colony formation of cervical cancer cells. **(A)** Structure of Cucurbitacin D. **(B)** Effect of Cucurbitacin D on cell viability of CaSki and SiHa cells. Cells (2500) were seeded in each well of 96-well plate and after overnight incubation, cells were treated with indicated concentrations of Cucurbitacin D for 72 h. Cell viability was assessed by MTS assay. The line graph represents the percent viable cells compared to the vehicle-treated cells. Each concentration value is the mean ± SE of triplicate wells of each group. **(C,D)** Effect of Cucurbitacin D on colony formation of cervical cancer cells. In brief, 250 cells were seeded in each well of 12-well plates. After 3 days, cells were treated with indicated concentrations of Cucurbitacin D and colonies obtained were stained with hematoxylin. Photographs were taken by UVP-gel documentation system for both CaSki (Ci) and SiHa (Di). Bar graphs represent number of colonies formed in each group of CaSki (Cii) and SiHa (Dii) cells. Experiments were repeated in triplicate with similar results. Asterisk (*) denotes the significant value p < 0.05 when applied student’s t-test.

To determine the effect of Cucurbitacin D (Fig. 1A) on proliferation of cervical cancer cells (CaSki and SiHa), MTS assay was performed. We observed that Cucurbitacin D treatment (0.05–1 μM) dose-dependently inhibited viability of cervical cancer cells. IC50 of Cucurbitacin D was 400 nM and 250 nM in CaSki and SiHa cells, respectively, after 72 hrs treatment (Fig. 1B). We next performed colony formation assay to investigate the long term treatment effect of Cucurbitacin D on proliferation of cervical cancer cells. In this experiment, CaSki and SiHa cells were treated with Cucurbitacin D at nanomolar concentrations (25 and 50 nM) for one week. Results demonstrated that Cucurbitacin D treatment significantly (p < 0.05) reduced the number of colonies formed in both CaSki (Fig. 1Ci,ii) and SiHa (Fig. 1Dii,iii) cells compared with respective controls. These findings suggest that Cucurbitacin D treatment inhibits the proliferation and clonogenic potential of cervical cancer cells.

### Cucurbitacin D inhibits invasion and migration of cervical cancer cells

**Figure 2:**
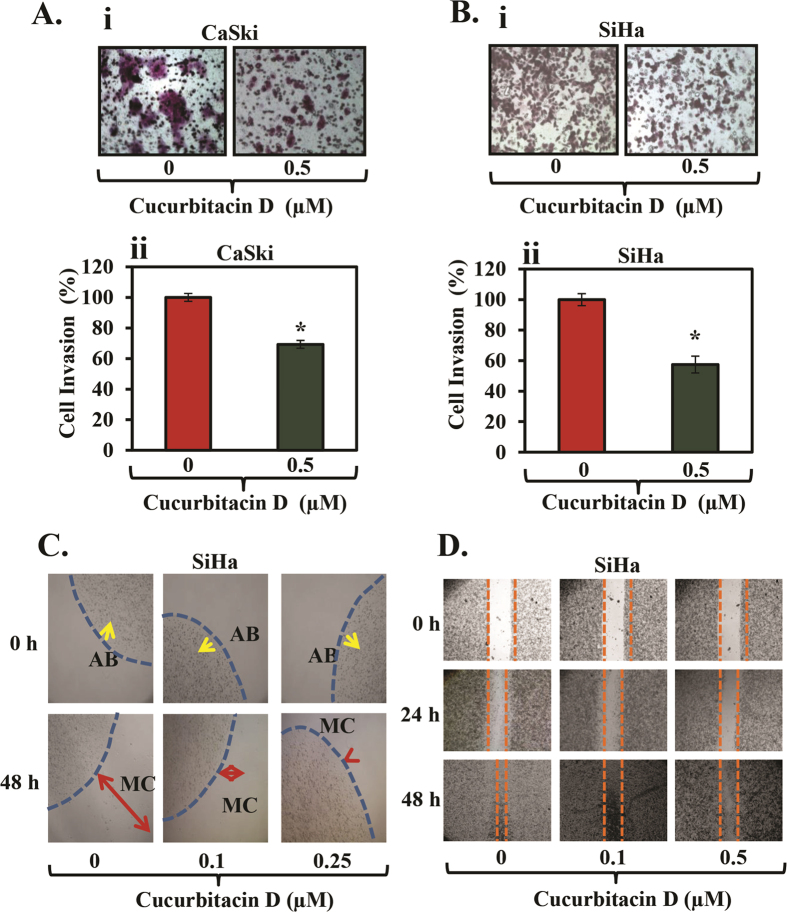
Effect of Cucurbitacin D on invasion and migration of cervical cancer cells. **(A,B)** Cucurbitacin D inhibits invasion of cervical cancer cells. Cell invasion assay was performed by a commercially available kit (BD Biosciences) as described in Materials and Methods. In brief, 18 hrs post-treatment of Cucurbitacin D, migrated cells were fixed, stained and counted. Representative images of invaded control and Cucurbitacin D treated SiHa (Ai) and CaSki (Bi) cells. Bar graphs represent the quantification of invaded SiHa (Aii) and CaSki (Bii) cells of control and Cucurbitacin D treated groups. Student’s t-test was performed to analyze significant difference. Asterisk (*) denotes the significant value p < 0.05. **(C,D)** Effect of Cucurbitacin D on cell motility of SiHa cells as determined by agarose bead and scratch wound assays. **(C)** Representative images of migratory cells (MC) in control and Cucurbitacin D treated groups at 0 and 48 hrs. AB denotes agarose bead. **(D)** Representative images of migratory SiHa cells in control and treated groups at 0, 24, and 48 hrs.

To determine the functional impact of Cucurbitacin D treatment on cervical cancer cells, we first performed *in vitro* cell invasion assay by using commercially available cell invasion kits. Our results illustrated that a dose of Cucurbitacin D (0.5 μM) effectively inhibited invasion of both CaSki (Fig. 2Ai,ii) and SiHa (Fig. 2Bi,ii) cells when compared with their respective control groups. We next performed bead assay to determine the effect of Cucurbitacin D on motility of CaSki and SiHa cells. Cucurbitacin D treatment effectively decreased the motility of SiHa cells (Fig. 2C) in a dose dependent manner. A similar effect was observed in CaSki cells (data not shown). Cucurbitacin D treatment also inhibited migration of SiHa cells (Fig. 2D) at 24 and 48 hrs as determined by scratch wound assay. These results strongly suggest that Cucurbitacin D has the potential to inhibit invasion and migration of cervical cancer cells.

### Cucurbitacin D induces apoptosis in cervical cancer cells

**Figure 3:**
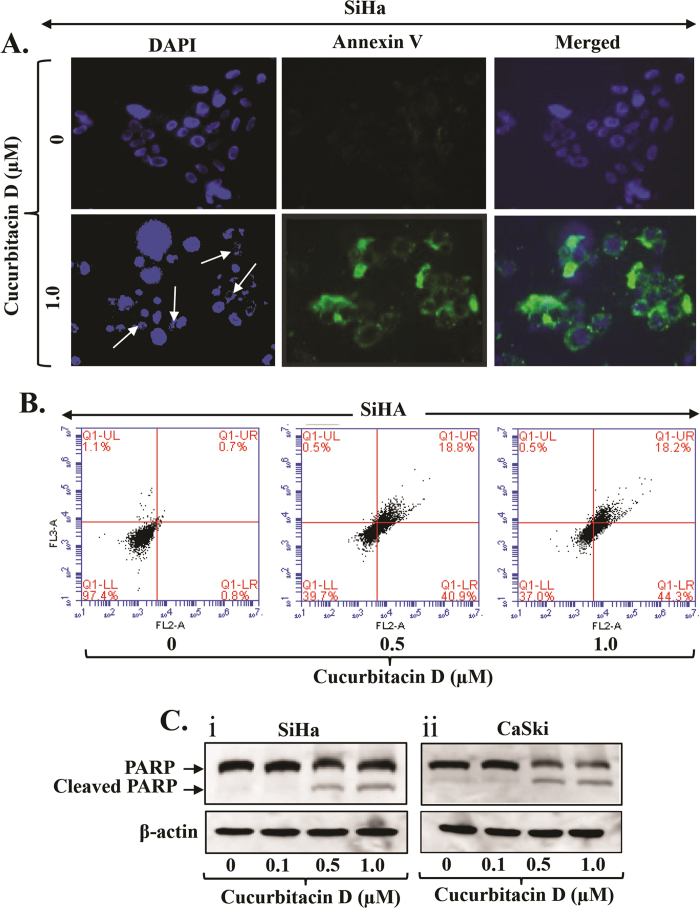
Effect of Cucurbitacin D on apoptosis in cervical cancer cells. **(A)**. Cucurbitacin D treatment induces apoptosis in cervical cancer cells as determined by DAPI and Annexin V staining. In brief, SiHa and CaSki cells were treated with indicated concentration of Cucurbitacin D for 24 hrs and apoptosis in these cells was measured by DAPI and Annexin V staining. Representative images of control and Cucurbitacin D treated SiHa (Ai) and CaSki (Aii) cells. Blue field represents DAPI staining while green field denotes Annexin V staining in the same focused area. White arrows indicate dysregulation of nucleus. **(B)** Quantification of early (Annexin V +ve) and late (7AAD +ve) apoptotic cells by flow cytometry in response to Cucurbitacin D treatment (0.5 and 1.0 μM) for 24 hrs. (**C)** Effect of Cucurbitacin D on total and cleaved PARP protein in both CaSki (Ci) and SiHa (Cii) cells as determined by Western blot analysis. Equal loading of protein in each lane was determined by stripping and probing the blot with β-actin antibody.

To elaborate our findings regarding the functional impact of Cucurbitacin D treatment on cervical cancer cells, we performed apoptosis assay. In this experiment, 70% confluent CaSki and SiHa cells were treated with Cucurbitacin D (1 μM) for 24 hrs and apoptosis induction was examined by DAPI and enhanced Annexin V staining by fluorescence microscopy as described in Materials and Methods. Cucurbitacin D treatment showed enhanced Annexin V staining and irregular nuclear morphology in both SiHa (Fig. 3A) and CaSki (Supplementary Fig. 8) cells. We further quantified the apoptosis by FACS analysis. Cucurbitacin D treatment (0.5–1.0 μM) of SiHa cells for 24 hrs resulted in dose-dependent increase of early apoptosis 41% and 44% respectively compared to control cells (Fig. 3B). However, Cucurbitacin D treatment of SiHa cells at 0.5 and 1.0 μM concentrations showed approximately similar trend in late-stage apoptosis (18%) (Fig. 3B). Cleavage in Poly (ADP-ribose) polymerase (PARP) protein is considered one of the biomarkers of early apoptosis. Thus, we examined the effect of Cucurbitacin D on total and cleaved PARP protein by Western blot analysis. Cucurbitacin D treatment illustrated a dose-dependent increase in the expression of cleaved PARP protein in both CaSki (Fig. 3Ci) and SiHa (Fig. 3Cii) cells, while the total PARP protein levels was inhibited which further confirms induction of apoptosis by Cucurbitacin D in cervical cancer cells.

### Cucurbitacin D treatment in cervical cancer cells arrests the cell cycle in G1/S phase

**Figure 4:**
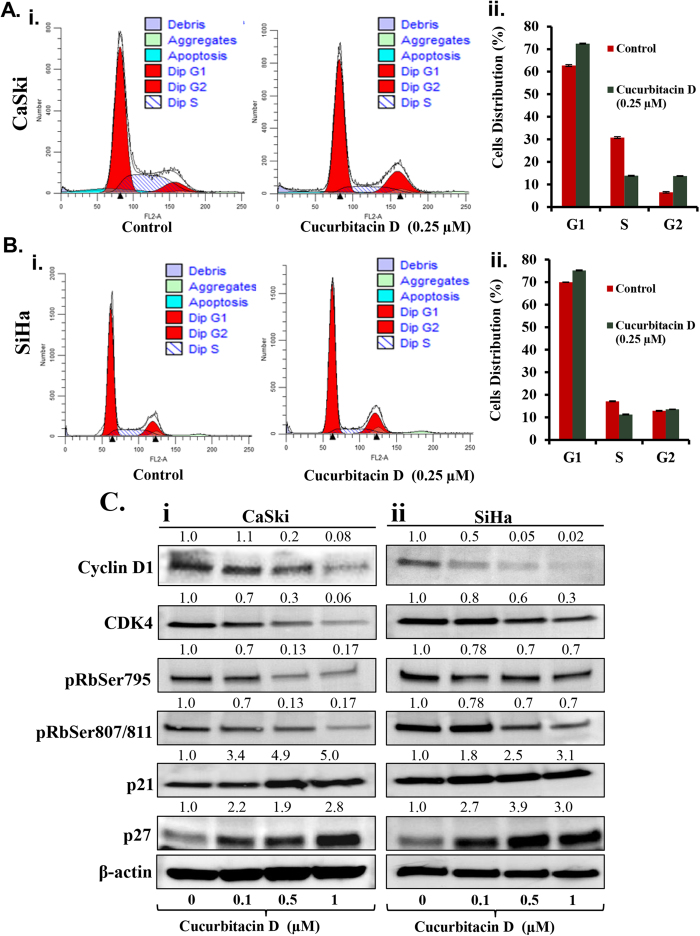
Effect of Cucurbitacin D on cell cycle progression in cervical cancer cells. Cucurbitacin D arrests CaSki and SiHa cell cycle in G1/S phase as determined by flow cytometry. (**A)** Histogram (i) and bar graph (ii) represent the cell cycle distribution in CaSki cells. **(B)** Histogram (i) and bar graph (ii) represent the cell cycle distribution in SiHa cells. (**C)** Effect of Cucurbitacin D on protein levels of cell cycle regulatory proteins (Cyclin D1, CDK4, pRbSer795, pRbser807/811, Rb, p21 and p27) in both CaSki (Ci) and SiHa (Cii) cells. Briefly, cells were treated with indicated concentrations of Cucurbitacin D for 24 hrs, total cell lysates were prepared and subjected for Western blot analysis. Equal loading of protein in each lane was determined by probing the blots with β-actin. Values shown above the blots are densitometric analysis of blots normalized with β-actin.

Cell cycle arrest has been appreciated as a target for the management of cancer^29^. To investigate the effect of Cucurbitacin D on distribution of cervical cancer cells in cell cycle, we performed cell cycle analysis by flow cytometry. Treatment of cells with 0.25 μM Cucurbitacin D resulted in an increase of CaSki (Fig. 4Ai,ii) and SiHa (Fig. 4Bi,ii) cells in G1/S phase of cell cycle, respectively, when compared with vehicle treated group. To further understand the molecular mechanism underlying cell cycle arrest, we examined the effect of Cucurbitacin D on cell-cycle regulatory proteins in cervical cancer cells. Western blot analysis demonstrated that Cucurbitacin D treatment inhibited cyclin D1 and cyclin dependent kinase 4 (CDK4) protein levels in both CaSki (Fig. 4Ci) and SiHa (Fig. 4Cii) cells. Cucurbitacin D treatment also inhibited phosphorylation of RB protein at 795 and 807/811 residues. We next examined the effect of Cucurbitacin D on cell cycle inhibitory proteins (p21 and p27). Western blot results demonstrated that Cucurbitacin D treatment dose-dependently increased the protein levels of p21 and p27 in both CaSki (Fig. 4Ci) and SiHa (Fig. 4Cii) cells compared to vehicle treated groups (Fig. 4Ci,ii).

### Cucurbitacin D treatment effectively inhibits oncogenic signaling pathways in cervical cancer cells

**Figure 5:**
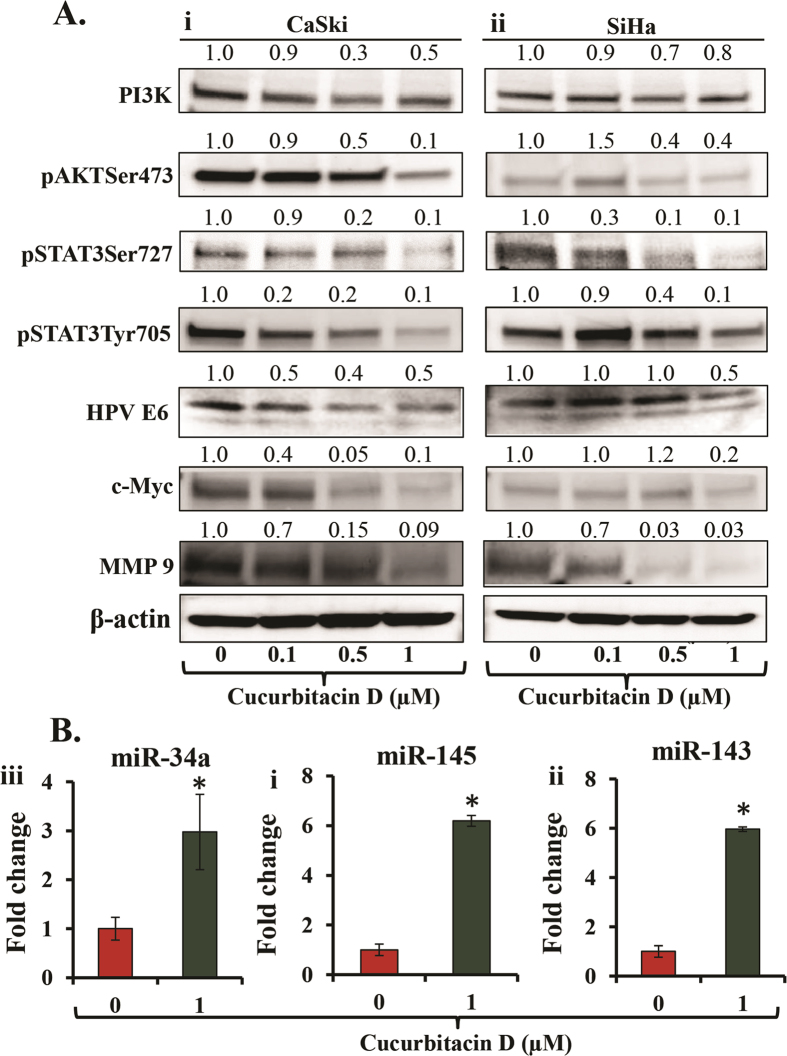
Effect of Cucurbitacin D on key oncogenic signaling proteins and tumor suppressor miRNAs in cervical cancer cells. (**A**) Cucurbitacin D inhibits the protein levels of PI3K, pAktSer473, pSTAT3Ser727, pSTAT3Ser705, c-Myc, and MMP-9 in CaSki (Ai) and SiHa (Aii) cells. Briefly, cells were treated with indicated concentrations of Cucurbitacin D for 24 hrs, total cell lysates were prepared and subjected for Western blot analysis. Equal loading of protein in each lane was determined by probing the blots with β-actin. Values shown above the blots are densitometric analysis of blots normalized with β-actin. (**B**) Effect of Cucurbitacin D treatment on the expression of miR-145 (Bi), miR-143 (Bii) and miR-34a (Biii) as determined by qRT-PCR analysis. RNU6B was used as an internal control. Student’s t-test was performed to analyze significant difference. Asterisk (*) denotes the significant value p < 0.01.

Several studies have shown that targeting PI3K/AKT pathways is an effective therapeutic approach against various types of cancer including cervical cancer. It has been shown that PI3K/AKT pathway is linked to cervical cancer cell proliferation and tumorigenesis^5^,^8^. Thus, we were interested to investigate the effect of Cucurbitacin D on PI3K/AKT signaling pathway in cervical cancer cells. Our results revealed that Cucurbitacin D treatment (0.1–1.0 μM) inhibited phosphorylation of AKT at Ser473 and PI3K110 in both CaSki (Fig. 5Ai) and SiHa (Fig. 5Aii) cells as determined by Western blot analysis. STAT3 is one of the oncogenic transcription factors constitutively activated in cervical cancer cells^6^. A recent study has suggested that STAT3 is involved in HPV-16-induced cervical carcinogenesis^30^. Thus, we examined the effect of Cucurbitacin D on STAT3 activation and expression of HPV-6 in cervical cancer cells. Western blot results demonstrated that Cucurbitacin D treatment (0.1–1.0 μM) dose-dependently inhibited phosphorylation of STAT3 at both Tyr705 and Ser727 residues in CaSki (Fig. 5Ai) and SiHa (Fig. 5Aii) cells. Cucurbitacin D treatment also inhibited protein level of HPV-6 in both Caski (Fig. 5Ai) and SiHa cells (Fig. 5Aii). It has been shown that constitutive activation of STAT3 induces proliferation and invasion of cancer cells *via* activation of several downstream target genes such as c-Myc and MMP9. Thus, we next examined the effects of Cucurbitacin D on these downstream target genes of STAT3. We found that Cucurbitacin D treatment (0.1 to 1 μM) also inhibited c-Myc and MMP9 protein levels in both CaSki (Fig. 5Ai) and SiHa (Fig. 5Aii) cells. These results suggest that Cucurbitacin D effectively inhibits PI3K/AKT and STAT3 signaling pathways in cervical cancer cells.

### Cucurbitacin D treatment induces the expression of miR-145, miR-143 and miR-34a in cervical cancer cells

Modulation of miRNAs has been reported during development, progression and metastasis of cervical cancer^31^. Expression of tumor suppressor miR-34a is inhibited in all stages of cervical cancer pathogenesis induced by either HPV16 or HPV18^32^,^33^. Our qRT-PCR result illustrated 3-fold induction of miR-34a expression in Cucurbitacin D treated (1 μM) CaSki cells (Fig. 5Bi) compared to vehicle control. Studies have also demonstrated that expression of miR-145 and miR-143 are significantly lower in cervical cancer when compared with their normal counterparts and over-expression of these miRNAs inhibit growth of cervical cancer cells^34^,^35^. Thus, we were interested to determine whether Cucurbitacin D treatment restores the expression of these tumor suppressor miRNAs in cervical cancer cells. Our results illustrated that treatment of CaSki cells with Cucurbitacin D (1 μM) significantly (p < 0.01) induced the expressions of miR-145 (Fig. 5Bii) and miR-143 (Fig. 5Biii) compared to control cells. These results suggest that Cucurbitacin D has potential to induce the expression of tumor suppressor miRNAs in cervical cancer cells.

### Cucurbitacin D inhibits cervical cancer cell-derived orthotopic xenograft tumors in athymic nude mice

**Figure 6:**
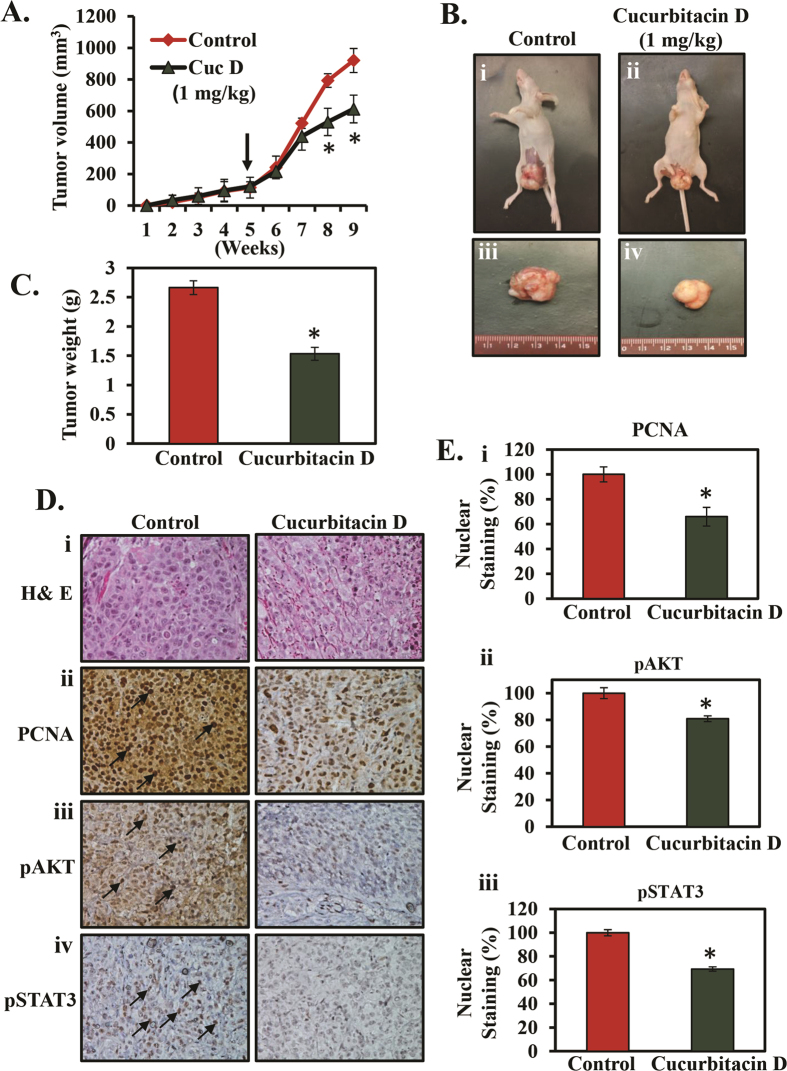
Effect of Cucurbitacin D on cervical cancer cell-derived orthotopic xenograft tumors in athymic nude mice. A total of 10 mice were used and divided into two groups: control (n = 5) and Cucurbitacin D (n = 5). CaSki cells (4 × 10^6^) were implanted into the cervix of each mouse. Cucurbitacin D (Cuc D) treatment (1 mg/kg body weight injected intra-tumorally 3 days a week) began 5 weeks after cell implantation and continued until 9 weeks. Control group mice received 0.2 ml PBS containing 0.1% DMSO. All mice were sacrificed at 9 weeks. (**A**) Line bar graph indicates regression of CaSki cell-derived orthotopic xenograft tumor volume in Cucurbitacin D treated mice compared to control group. Values in bar graph represent mean ± SE of five mice tumors in each group. (**B)** Representative photographs of athymic nude mice bearing CaSki cell-derived orthotopic xenograft tumor in control (Bi) and Cucurbitacin D treated mice (Bii). Representative images of excised orthotopic xenograft tumors of control (Biii) and Cucurbitacin D treated (Biv) mice. **(C)** Bar graph indicates weight of excised tumors of control and Cucurbitacin D treated mice. Values in graph show mean±SE of five tumors. (**D)** Representative H&E staining images of control and Cucurbitacin D treated excised tumors (Di). Effects of Cucurbitacin D on the expression of PCNA (Dii), pAKT (Diii), and pSTAT3 (Div) in excised tumor tissues of control and Cucurbitacin D treated mice as determined by immunohistochemistry. (**E)** Bar graphs represent quantification of immunohistochemistry images of PCNA (Ei), pAKT (Eii), and pSTAT3 (Eiii). Student’s t-test was performed to analyze significant difference. Asterisk (*) denotes the significant value p < 0.05.

To determine whether Cucurbitacin D treatment inhibits cervical tumor growth, we conducted an orthotopic xenograft study. In this experiment, n = 10 athymic nude mice were used to establish cervical cancer cell-derived orthotopic xenograft tumors and were divided into two groups as described in Materials and Methods. Our results illustrated that intra-tumoral administration of Cucurbitacin D (1 mg/kg body weight) significantly (p < 0.01) inhibited CaSki cell-derived orthotopic xenograft tumors in athymic nude mice compared to vehicle treated mice tumors (Fig. 6A–C). As shown in Fig. 6A, Cucurbitacin D administration (1 mg/kg body weight) significantly (p < 0.05) reduced both tumor volume and weight compared to control group’s mice. To determine whether Cucurbitacin D inhibits the phosphorylation of STAT3 and AKT in *in vivo* condition, we performed IHC of pAKT and pSTAT3 in excised xenograft tumor tissues of control and Cucurbitacin D administered mice. Our results illustrated significant decreased expression of pAKT and pSTAT3 proteins when compared with control group (Fig. 6D). Proliferative cell nuclear antigen (PCNA) is considered one of the markers of cell proliferation. Thus, we examined the effect of Cucurbitacin D on the expression of PCNA. Our results showed a marked decrease in the expression of PCNA in the nucleus of Cucurbitacin D treated mice xenograft tumors compared with control (Fig. 6D). These results indicate potential anti-tumor efficacy of Cucurbitacin D against cervical cancer.

## Discussion

Cervical cancer is a leading cause of cancer related deaths in women worldwide including US (1). HPV infection is the major risk factor for the prevalence of cervical cancer. Among all HPVs, HPV-16 and HPV18 are considered to be responsible for 70% of cervical cancer pathogenesis^2^. Although chemo-radiotherapy and surgery can cure 80–95% of women with early-stage cervical cancer, recurrent and metastatic disease remains a major cause of cervical cancer death in patients. Several therapeutic strategies such as vaccination or use of various chemotherapeutic drug combinations have shown success in the prevention and treatment of cervical cancer. However, both vaccinations and the use of these chemotherapeutic drugs have some limitations because of their toxicity. These limitations can be overcome by utilizing complementary and alternative medicine. Plant derived natural products have gained a lot of attention because they are less toxic compared to current chemotherapeutic drugs^36^. In this study, we provide evidence of the potential therapeutic efficacy of Cucurbitacin D (one of the novel analogues of cucurbitacin) against cervical cancer using HPV-16 positive human cervical cancer cells (CaSki and SiHa) as a model system. The result of our functional studies indicate that Cucurbitacin D has potential to inhibit the growth and proliferation of cervical cancer cells.

Invasion and migration are hallmarks of the metastatic characteristics of a cancer cell. Thus, agents which inhibit invasion and migration of cancer cells could be used for the prevention and treatment of metastatic cancer. Our functional experiment shows that non-toxic doses of Cucurbitacin D significantly (p < 0.01) inhibit invasion and migration of cervical cancer cells, which indicate that Cucurbitacin D could be an effective agent to inhibit cervical cancer cell metastasis. However, a detailed study is warranted to investigate the effect of Cucurbitacin D treatment on metastatic growth of cervical cancer cells in an appropriate mouse model of cervical cancer.

Agents that induce apoptosis can be ideal candidates for cancer therapy. Various plant-derived agents including cucurbitacin derivatives have been shown to induce apoptosis in various cancer cells^37^,^38^. Our results of enhanced Annexin V staining and cleavage of PARP protein in cervical cancer cells demonstrate that Cucurbitacin D is an apoptosis-inducing agent. Various studies have shown that agents responsible for inducing cell cycle arrest in G1 phase could be used for the treatment of various types of cancer including cervical cancer^29^. G1 abrogation of the cell cycle prevents cancer cells from repairing DNA and inhibits them from entering the S phase. Thus, the G1 checkpoint has emerged as an attractive therapeutic target for cancer therapy. Our results indicate that Cucurbitacin D exerted a strong growth inhibitory effect on cervical cancer cells by arresting cells in G1/S phase of cell cycle. It is known that cell cycle is primarily regulated by cyclins and cyclin-dependent kinases (CDKs), which are critical for the progression of the cell cycle; their inactivation leads to cell cycle arrest^39^. The observed inhibitory effects of Cucurbitacin D on cyclin D1, CDK4 and phosphorylation of RB proteins in cervical cancer cells suggests its interference in cell cycle regulatory proteins. CDK activity is regulated by CDK inhibitors such as the p21/WAF1 and p27/KIP1 families of proteins. Our results indicate that Cucurbitacin D induces the expression of both p21 and p27 protein levels in cervical cancer cells. All of these results suggest that Cucurbitacin D has potential in arresting the G1/S phase *via* modulating cell cycle regulatory proteins to inhibit the growth of cervical cancer cells.

Various oncogenic signaling molecules are involved in the induction, progression and metastasis of cervical cancer^40^. PI3K/AKT is one of the important signaling pathways that has been shown to be linked with progression and metastasis of cervical cancer^8^. Studies have shown that inhibition of the PI3K/AKT signaling pathway in cervical cancer cells induce apoptosis. Our results show the inhibition of PI3K and pAKTSer473 protein levels in cervical cancer cells, which suggest that Cucurbitacin D has potential to inhibit PI3K/AKT signaling pathways in cervical cancer cells. STAT3 is one of the transcription factors which regulates PI3K/AKT signaling pathways and is involved in poor prognosis of cervical cancer^4^,^5^,^6^,^7^,^8^. A cervical cancer tissue microarray study has shown that constitutive activation of STAT3 correlates with the progression of HPV positive cervical cancer^41^. Another study from same group has shown the functional role of STAT3 in HPV16 induced cervical carcinogenesis^30^. Our results clearly indicate that Cucurbitacin D targets both STAT3 and HPV E6 in cervical cancer cells. It may be possible that Cucurbitacin D may inhibit HPV E6 oncoprotein directly or indirectly through STAT3. Further studies are required in this direction. Cucurbitacin treatment also inhibited downstream target genes of STAT3 such as c-Myc, and MMP9. These results further confirm that STAT3 is one of the potential molecular targets of Cucurbitacin D in cervical cancer cells. These findings correlate with previously published reports of cucurbitacin effects on STAT3 in various cancer cells^38^.

It has been reported that several miRNAs are modulated during the development, progression and metastasis of various cancers including cervical cancer^31^. These modulated miRNAs in cancer can function as oncogenes or tumor suppressor genes. Studies have reported that miR-145 and miR143 are significantly lower in cervical cancer and expression of these miRNA inhibits growth of cervical cancer cells^34^,^35^. Our data show a significant (p < 0.01) increase of miR-145 and miR-143 in cervical cancer cells after treatment with Cucurbitacin D, which clearly indicates that Cucurbitacin D may inhibit the growth of cervical cancer cells *via* inducing the expression of tumor suppressing miR-145 and miR-143. It has been reported that miR34a is the direct transcriptional target of p53^33^. Since HPV E6 oncoproteins destabilize p53 proteins during virus infection, it is assumed that miR34a expression can be downregulated in most cervical cancer tissues infected with HPV infection. Thus, Cucurbitacin D may induce the expression of p53 via restoration of miR-34a in cervical cancer cells.

Because Cucurbitacin D treatment showed growth inhibition of cervical cancer cells under *in vitro* condition, we asked whether these data could be translated to an *in vivo* situation. Our tumor xenograft study showed that intra-tumoral administration of Cucurbitacin D (1 mg/kg body weight) inhibited cervical cancer cell-derived orthotopic xenograft tumors in athymic nude mice. We did not observe any apparent toxicity in any of the Cucurbitacin D administered mice. These results clearly indicate that a dose of 1 mg/kg body weight of Cucurbitacin D has potential to inhibit human cervical cancer cell-derived xenograft tumors without any toxic side effects. Cucurbitacin D administration showed significant (p < 0.01) decreased expression of PCNA protein levels in excised xenograft tumors tissues, which further indicates that Cucurbitacin D has potential to inhibit proliferation of cervical tumor cells.

## Conclusion

Our study indicates for the first time that Cucurbitacin D has the potential to inhibit the growth of HPV16 positive cervical cancer cells *in vitro* and *in vivo via* inhibiting key oncogenic signaling pathways and inducing tumor suppressor miRNAs. Thus, Cucurbitacin D could be a useful therapeutic agent for the treatment of cervical cancer.

## Materials and Methods

### Cell lines

HPV-16 positive human cervical cancer cells (CaSki and SiHa) were purchased from American Type Cell Culture (Manassas, VA) and cultured in RPMI-1640 and DMEM medium (HyClone Laboratories, Inc., Logan, UT) supplemented with 10% heat-inactivated FBS (Atlanta Biologicals, Norcross, GA) and 1% antibiotics (penicillin and streptomycin).

### Chemicals and antibodies

Specific monoclonal and polyclonal antibodies of β-actin (cat. # 3700), PARP (cat. # 9542S), cyclin D1 (cat. # 2922), CDK4 (cat. # 12790), pRbSer 795 (cat. # 9301), pRb807/811 (cat. # 9308), p21 (cat. # 2947), p27 (cat. # 3686), PI3K110 (cat. # 4249), pAKT (cat. # Ser473) (cat. # 4060), pSTAT3Ser727 (cat. # 9145), pSTAT3Tyr705 (cat. # 9136), STAT3 (cat. # 9139) and MMP9 (cat. # 13667) were obtained from Cell Signaling Technology Inc. HPV E6 (cat. # AB70) specific antibody was procured from Abcam Cambridge, MA. Cucurbitacin D was obtained from Dr. Fathi T. Halaweish (SDSU, Brookings, SD). Detailed procedure for synthesis and characterization of Cucurbitacin D was described^17^.

### MTS assay

To determine the effect of Cucurbitacin D on cell proliferation of CaSki and SiHa cells, MTS assay was performed. Briefly, cells were seeded at a density of 2.5 × 10^3^ cells per well in 96-well plates and incubated overnight in a CO_2_ incubator. Cucurbitacin D was dissolved in DMSO and diluted in culture media. The cells were treated with various concentrations of Cucurbitacin D (0.05, 0.1, 0.25, 0.5 and 1 μM) for 72 hrs. Control group cells were treated with 0.01% DMSO in culture media. Cell viability was determined by adding 20 μL of 3-(4,5-Dimethylthiazol-2-yl)-5-(3-carboxymethoxyphenyl)-2-(4-sulfophenyl)-2H-tetrazolium, inner salt (MTS) reagent, incubating for 2 hrs and measuring the absorption at 490 nm using a SPECTRA Max Plus Plate Reader (Molecular Devices, Sunnyvale, CA). The percent cell viability in treated cells was calculated by normalizing the cells with 100% viable vehicle treatment group.

### Colony formation assay

In this experiment, CaSki or SiHa cells (250) were seeded in a 12-well plate and allowed to stand for the next three days. Cells were treated with Cucurbitacin D (25 and 50 nM) for one week. Control cells were treated with 0.1% DMSO. The cells were maintained under standard cell culture conditions at 37 °C and 5% CO_2_ in a humid environment. Colonies were fixed in methanol, stained with haematoxylin, and counted using UVP 810 software.

### Chemoinvasion assay

Cell invasion assay was performed using a cell invasion kit (BD Biocoat^TM^ Matrigel Invasion Chambers; BD Biosciences, San Jose, CA)^42^. All procedures were followed as per the manufacturer’s instructions. In brief, CaSki or SiHa cells (50,000 cells/well) were seeded in an upper chamber containing serum free medium and further treated with Cucurbitacin D(0.5 μM) or 0.1% DMSO as vehicle for 24 hrs. The lower chamber was filled with 500 μL of media containing 20% FBS. Twenty-four hrs post-treatment, cells were completely removed from inside the upper chamber by cotton swab. Cells were fixed with methanol and stained with Crystal Violet. Invaded cells were observed by using a light microscope at 100X magnification. Cells that had invaded the matrix membrane were counted in three random fields of view and the experiments were performed in triplicate.

### Wound healing assay

Cervical cancer cell migration was assessed by an *in vitro* wound-healing assay as previously described^43^. Briefly, cells were seeded in a 12-well plate and after 80–90% confluency, a standardized wound was made using a 200 μL micropipette tip. Cells were treated with Cucurbitacin D (0.1, 0.5, 1 μM) and photographed at 24 and 48 hrs by phase contrast microscopy. Motility of the cells was measured by their ability to close the wound.

### Agarose bead assay for cell motility

Cellular motility was determined by an agarose bead-based cell motility assay as described earlier^43^. Briefly, cells were mixed into a low melting point agarose solution and drops of suspension were placed onto plates. At 24 and 48 hrs the plates were photographed using a phase-contrast microscope.

### Detection of apoptosis

The apoptosis inducing effect of Cucurbitacin D on cervical cancer cells was analyzed by Annexin V-FLUOS staining kit (Roche Diagnostic Corp., Indianapolis, IN). In this experiment, 60% confluent CaSki and SiHa cells were treated with Cucurbitacin D for 24 hrs. Cells were washed with PBS (1X) and kept in DAPI and Annexin-V solution for 20 min. Images were captured under fluorescence microscope. Apoptosis was further determined by Annexin V-7AAD kit (BD Biosciences). In this experiment, SiHa cells (1 × 10^6^) were plated in a 100 mm dish and allowed to adhere overnight. The next day, cells were treated with 0.5 and 1.0 μM Cucurbitacin D or equivalent amounts of controls for 24 hrs. Both adherent and floating cells were collected and stained with Annexin V and 7-AAD (BD Biosciences) at 5 μL of each/100 μL of cell suspension. Cells were incubated for 20 min in the dark at room temperature and analyzed with an Accuri C6 Flow Cytometer in FL2 and FL3 channels.

### Cell cycle analysis

In this experiment, approximately 70% confluent CaSki and SiHa cells were treated with Cucurbitacin D (250 nM) for 24 hrs. The cells were trypsinized and washed twice with ice cold PBS (1X). The cell pellet was resuspended in 50 μL ice cold PBS (1X) and 450 μL cold methanol and left for 1 hr at 4 °C. The cells were washed twice with ice cold PBS (1X), suspended in 500 μL PBS, and incubated with 5 μL RNase (20 μg/ml final concentration) at 37 °C for 1 hr. The cells were chilled over ice for 10 min and stained with propidium iodide (50 μg/ml final concentration for 1 hr and analyzed by flow cytometry (BD Accuri C6; Becton-Dickinson, Mountain View, CA).

### Western blot analysis

To determine the effects of Cucurbitacin D on oncogenic protein levels in cervical cancer cells, we performed Western blot analysis. In these experiments, 70–80% confluent CaSki and SiHa cells were treated with Cucurbitacin D (0.1, 0.5, and 1 μM) for 24 hrs. Control cells were treated with vehicle (0.1% DMSO). Total cell lysate was prepared using lysis buffer (50 mM N-2-hydroxyethylpiperazine-N’-2-ethanesulfonic acid [HEPES, pH 7.5], 150 mM NaCl, 10% glycerol, 1% Triton X-100, 1.5 mM MgCl_**2**_, 10 μg/ml aprotinin, 10 μg/ml leupeptin, 1 mM phenylmethylsulfonyl fluoride [PMSF], 200 mM Na_**3**_VO_4_, 200 mM NaF and 1 mM EGTA). Cells were washed with PBS (1X), collected in lysis buffer and left on ice for 30 min, sonicated and centrifuged at 13,000 rpm for 30 min at 4 °C. Supernatants were collected and stored at −80 °C until use for Western blot analysis. Thirty micrograms of protein of each group was fractionated on 4–10% SDS-polyacrylamide gels (Bio-Rad, Hercules, CA). The proteins were transferred to Hybond-P polyvinylidene difluoride (PVDF) transfer membrane (Amersham, Pittsburgh, PA). The membranes were then incubated with the indicated primary antibodies followed by a horseradish peroxidase secondary antibody and developed with enhanced chemiluminescence reagent (Roche) using a UVP gel documentation system. The Western blots were quantitated by densitometry analysis using gelQuant software.

### Quantitative real-time PCR (qRT-PCR)

To investigate the effect of Cucurbitacin D treatment on the expression of miRNAs (miR-145, miR-143 and miR-34a) in cervical cancer cells, we performed qRT-PCR. In brief, approximately 70% confluent CaSki cells were treated with Cucurbitacin D (1.0 μM) and control cells were treated with 0.01% DMSO for 24 hrs. Total RNA was extracted using Trizol reagent (Invitrogen, Life Technologies, Grand Island, NY). The integrity of the RNA was measured with an RNA 6000 Nano Assay kit and 2100 Bioanalyzer (Agilent Technologies, Santa Clara, CA). For miRNA detection, 100 ng total RNA was reverse transcribed into cDNA using specific primers designed for miRNA analysis (Applied Biosystems, Foster City, CA). The expression of these miRNAs was determined by qRT-PCR using the Taqman PCR master mixture (no AmpErase UNG) and specific primers designed for detection of mature miRNAs (Applied Biosystems). The expression of miRNA was normalized with the expression of endogenous control, U6snRNA.

### Orthotopic xenograft study

Six-week-old female athymic nude mice were obtained from Jackson Laboratory (Bar Harbor, ME) to generate an orthotopic mouse model of cervical cancer. The mice were maintained in a pathogen-free environment and all the procedures were carried out as per the protocol approved by the UTHSC Institutional Animal Care and Use Committee (UTHSC-IACUC). A total of 10 mice were used in the study. Briefly, CaSki cells (4 × 10^6^) were dispersed in 100 μL PBS (1X) and 100 μL Matrigel (BD Biosciences) and injected directly into the cervix of each mouse without any surgery. The mice were periodically monitored for tumor development and tumor volume was measured from one week after injection using a digital Vernier caliper. When the tumor volume reached ~200 mm^3^, Cucurbitacin D (1 mg/kg) and the respective vehicle control (1X PBS) were injected intra-tumorally three times per week for 4 weeks. The tumor volume was calculated using the ellipsoid volume formula: tumor volume (mm^3^) = *0.5* × *L* × *W* × *H*, wherein *L* is length, *W* is width, and *H* is height. The tumor was regularly monitored and allowed to grow until the tumor burden of control group mice reached a targeted volume of 1000 mm^3^. Mice were sacrificed and their tumors were excised and used for tissue sectioning (5 micron), histopathology and lysate preparations.

### Immunohistochemistry (IHC)

IHC analysis for PCNA, pAKT, and pSTAT3 proteins was performed on formalin fixed, paraffin embedded orthotopic xenograft tumors (5 micron sections). Briefly, the tumor tissues were deparaffinized, rehydrated, treated with 0.3% hydrogen peroxide and processed for antigen retrieval using a heat-induced technique. Following blocking with background sniper (Biocare Medical, Concord, CA), the samples were processed for staining with PCNA, pAKT, and pSTAT3 antibodies (Cell Signaling Technologies, Danvers, MA). The expression of these proteins was detected using a MACH 4 Universal HRP Polymer detection kit (Biocare Medical) and 3, 9-diaminobenzidine (DAB substrate kit, Vector Laboratories, Burlingame, CA). The slides were counterstained with hematoxylin, dehydrated, mounted with VectaMount (Vector Laboratories) and visualized using an Olympus BX 41 Microscope (Olympus Corporation, Tokyo, Japan).

### Statistical analysis

Statistical analysis was performed using an unpaired two-tailed Student *t*-test and employed to assess the statistical significance between the control and Cucurbitacin D- treated groups. p value  < 0.05 was considered as significant.

## Additional Information

**How to cite this article**: Sikander, M. *et al*. Cucurbitacin D exhibits potent anti-cancer activity in cervical cancer. *Sci. Rep.* **6**, 36594; doi: 10.1038/srep36594 (2016).

**Publisher’s note:** Springer Nature remains neutral with regard to jurisdictional claims in published maps and institutional affiliations.

## Supplementary Material

Supplementary Information
