# Supplementary material for: Cucurbitacin D exhibits potent anti-cancer activity in cervical cancer
Source: Sci Rep. 2016 Nov 8;6:36594. doi: 10.1038/srep36594 (PMC5100479; doi:10.1038/srep36594)
Supplement: Supplementary Information [file srep36594-s1.pdf]

Cucurbitacin D exhibits potent anti-cancer activity in cervical cancer

†Mohammed Sikander[a], †Bilal Bin Hafeez[a], Shabnam Malik [a], Abdulrhman Alsayari[b], Fathi T. Halaweish[c], Murali M. Yallapu[a], *Subhash C. Chauhan[a], *Meena Jaggi[a]

[a]University of Tennessee Health Science Centre, Memphis, TN, USA, 38163,
[b]King Khaled University, College of Pharmacy, Box 188, Abha, 61441, Saudi Arabia
[c]South Dakota State University, Brookings, SD, USA, 57007

† Contributed equally to this work

Conflict of interest: None

*Correspondence and requests for materials should be addressed to MJ.

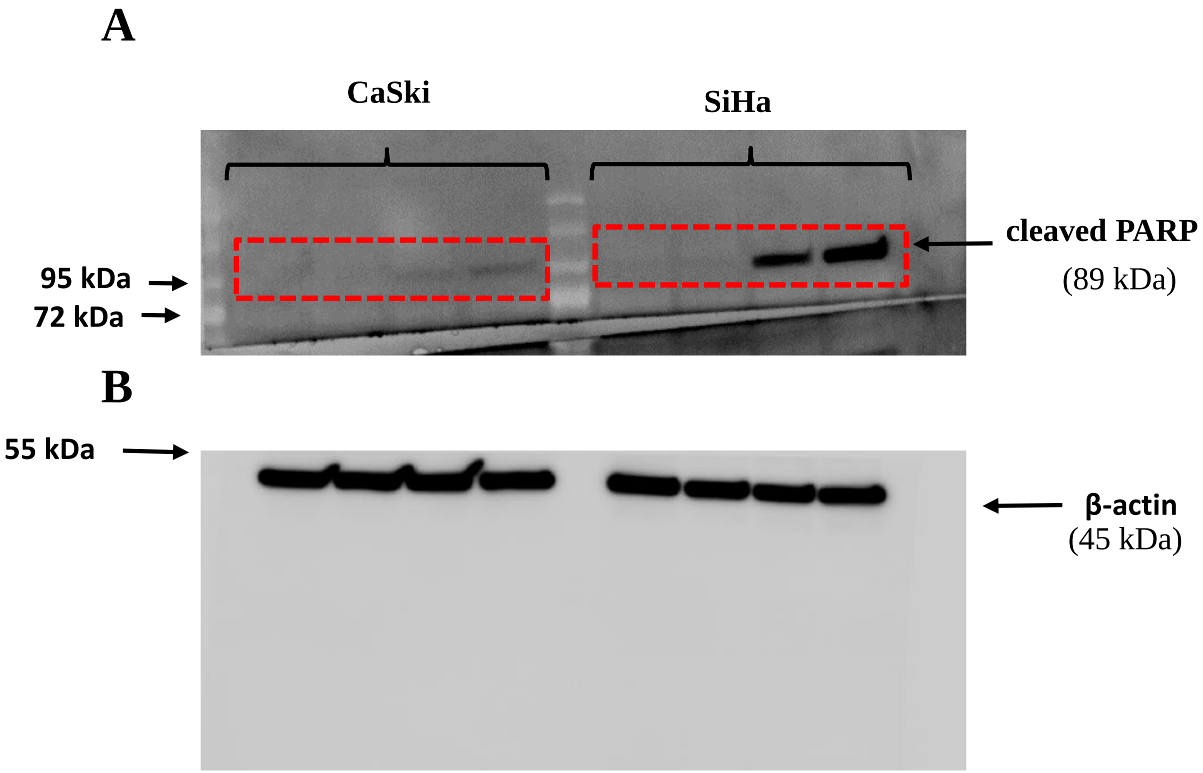

Supplementary Figure 1: A. Western blots of cleaved PARP Protein as shown in Fig. 3C. This blot was cut from 55 kDa. B. Western blot of β-actin shown in Fig. 3C.

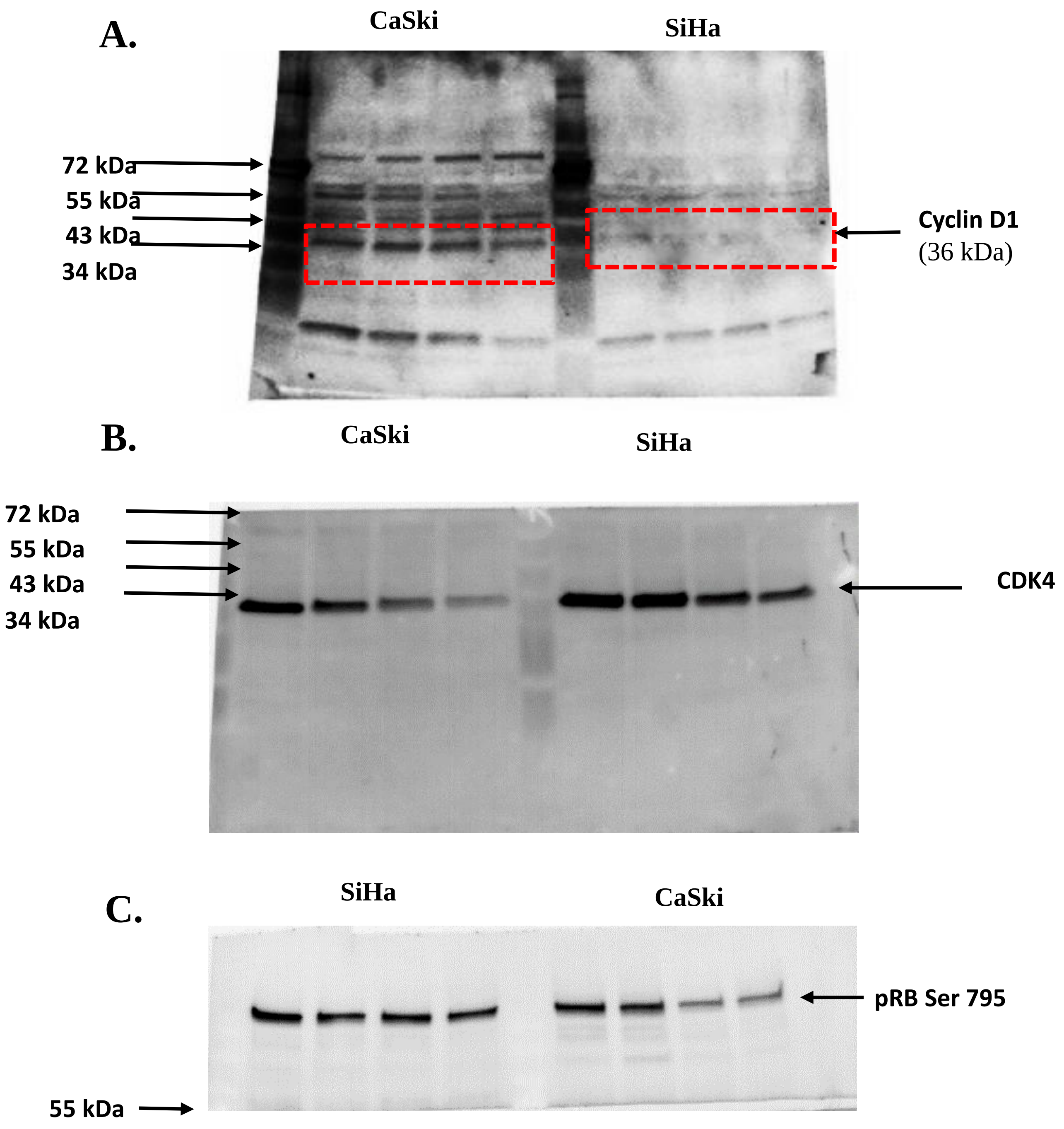

Supplementary Figure 2: Western blot of Cyclin D1, CDK4, and pRBSer795 in both CaSki and SiHa cells as shown in Fig. 4B. pRBSer795 blot was cut from 55 kDa to determine other protein.

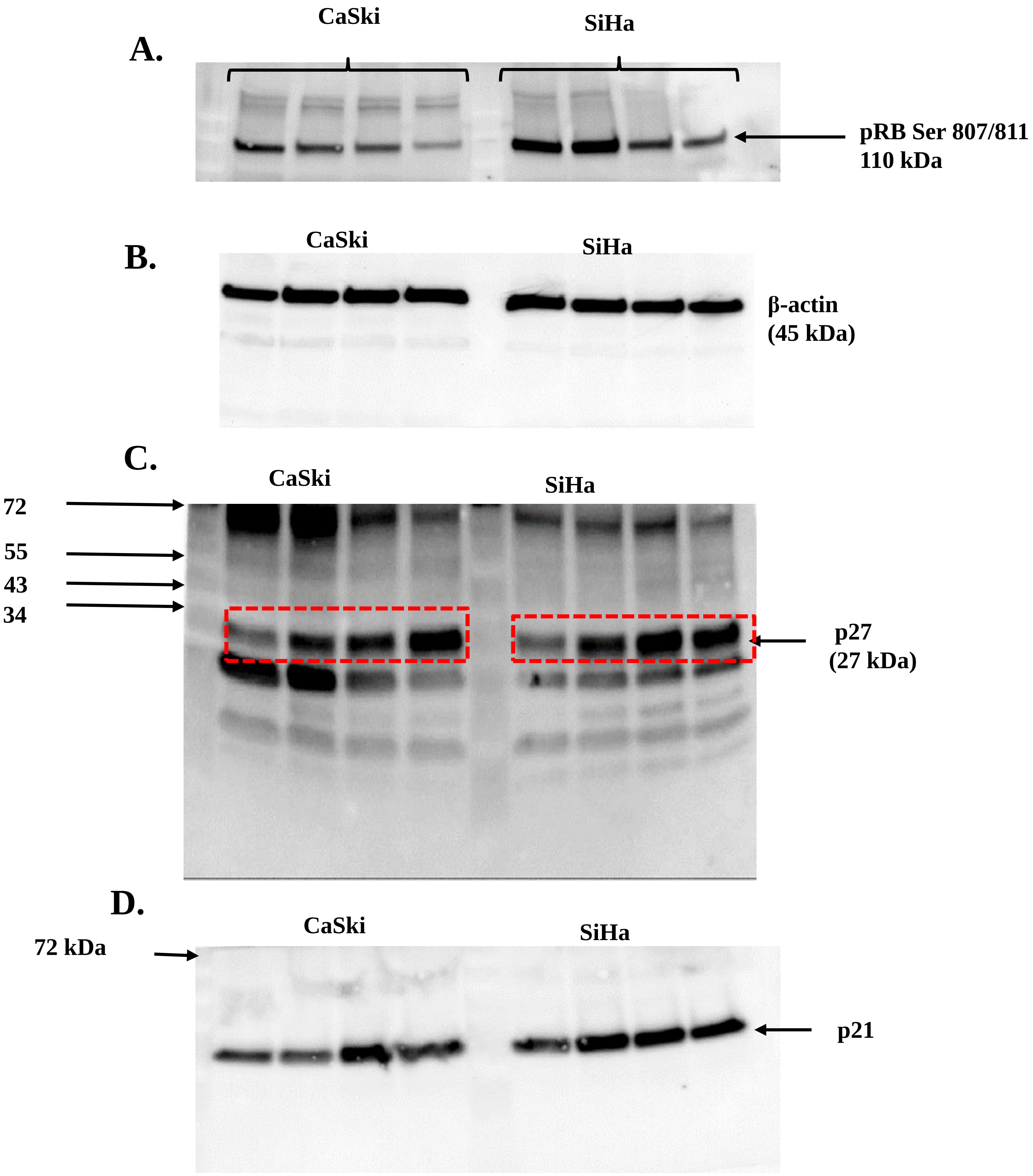

Supplementary Figure 4: A. Western blot of pRBSer7807/811. This blot was cut at 72 kDa to detect other protein. B. β-actin blot of Fig. 4B. C. Western blot of p27 shown in Fig. 4B. This blot was cut at 72 kDa. D. p21 blot shown in Fig 4B.

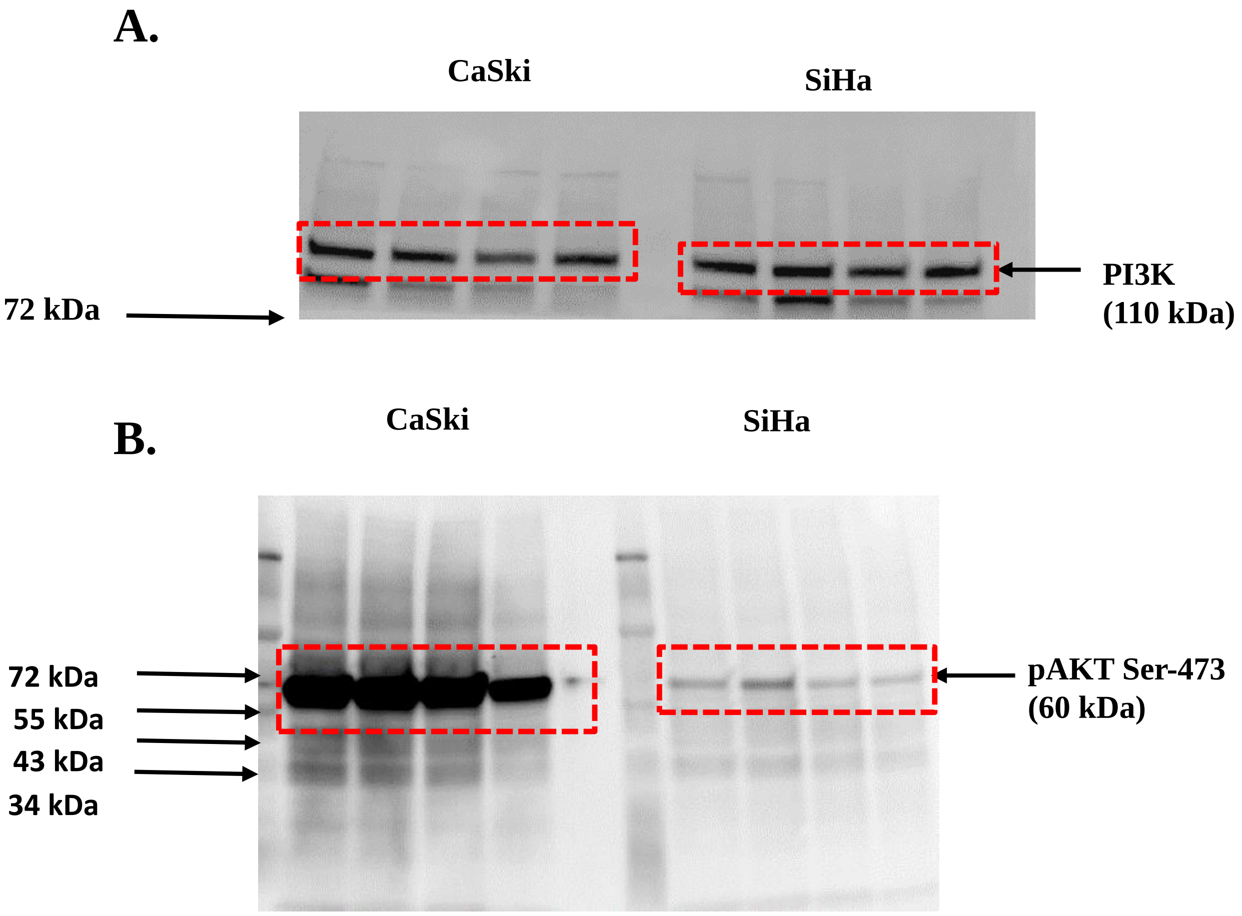

Supplementary Figure 5:. **A.** Western blot of PI3K110 (catalytic subunit) as shown in Fig. 5A. This blot was cut at 72 kDa to detect other low molecular weight protein. **B.** Full blot of PhosphoAKT Ser-473 as shown in Fig.5.

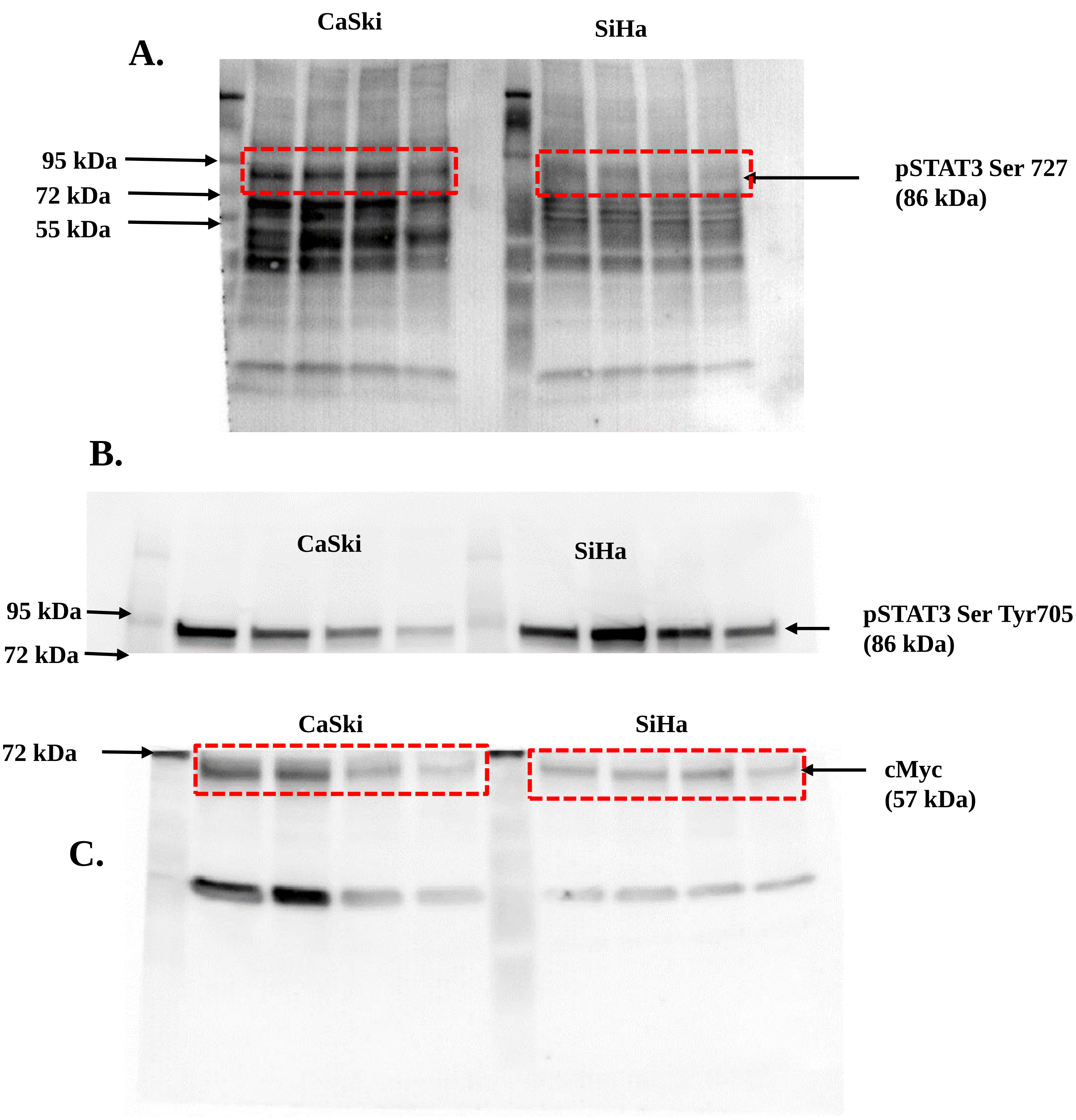

Supplementary Figure 6: A. Full blot of pSTAT3Ser727 as shown in Fig. 5. **B.** Blot showing pSTAT3 Tyr705. This blot was cut at 72 kDa to detect other lower molecular weight protein (Shown in Fig 5). **C.** CMyc blot shown in Fig. 5. This blot was cut at 72 kDa to detect higher molecular weight protein.

A

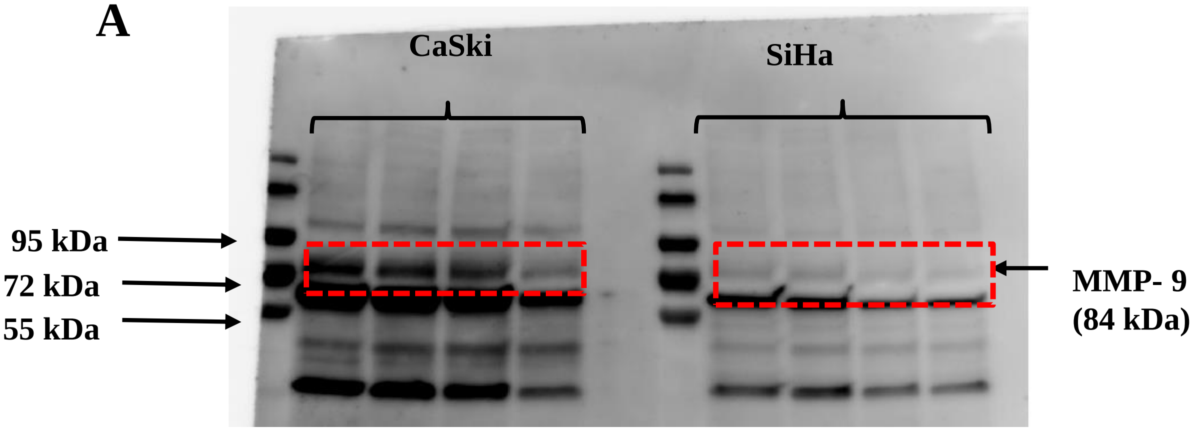

B

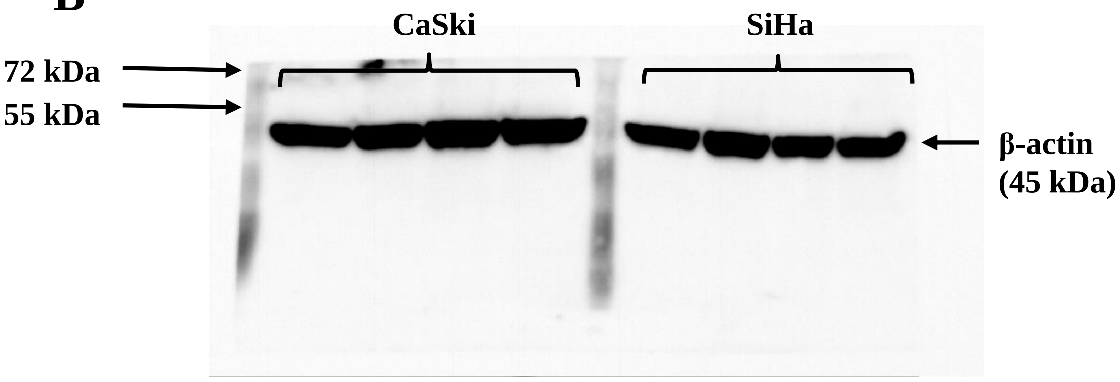

Supplementary Figure 7: A. Full blot of MMP9 as shown in Fig. 5 A. **B.** β-actin blot of Fig. 5A.

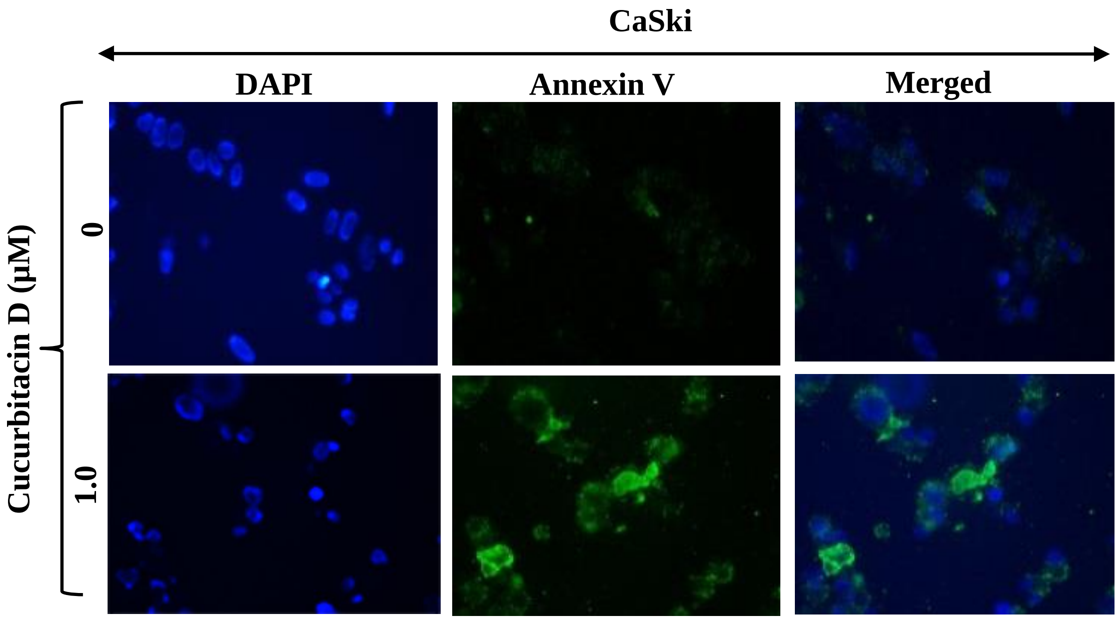

Supplementary Figure 8A: Effect of Cucurbitacin D on apoptosis of CaSki cells as determined by Annexin V and DAPI staining.

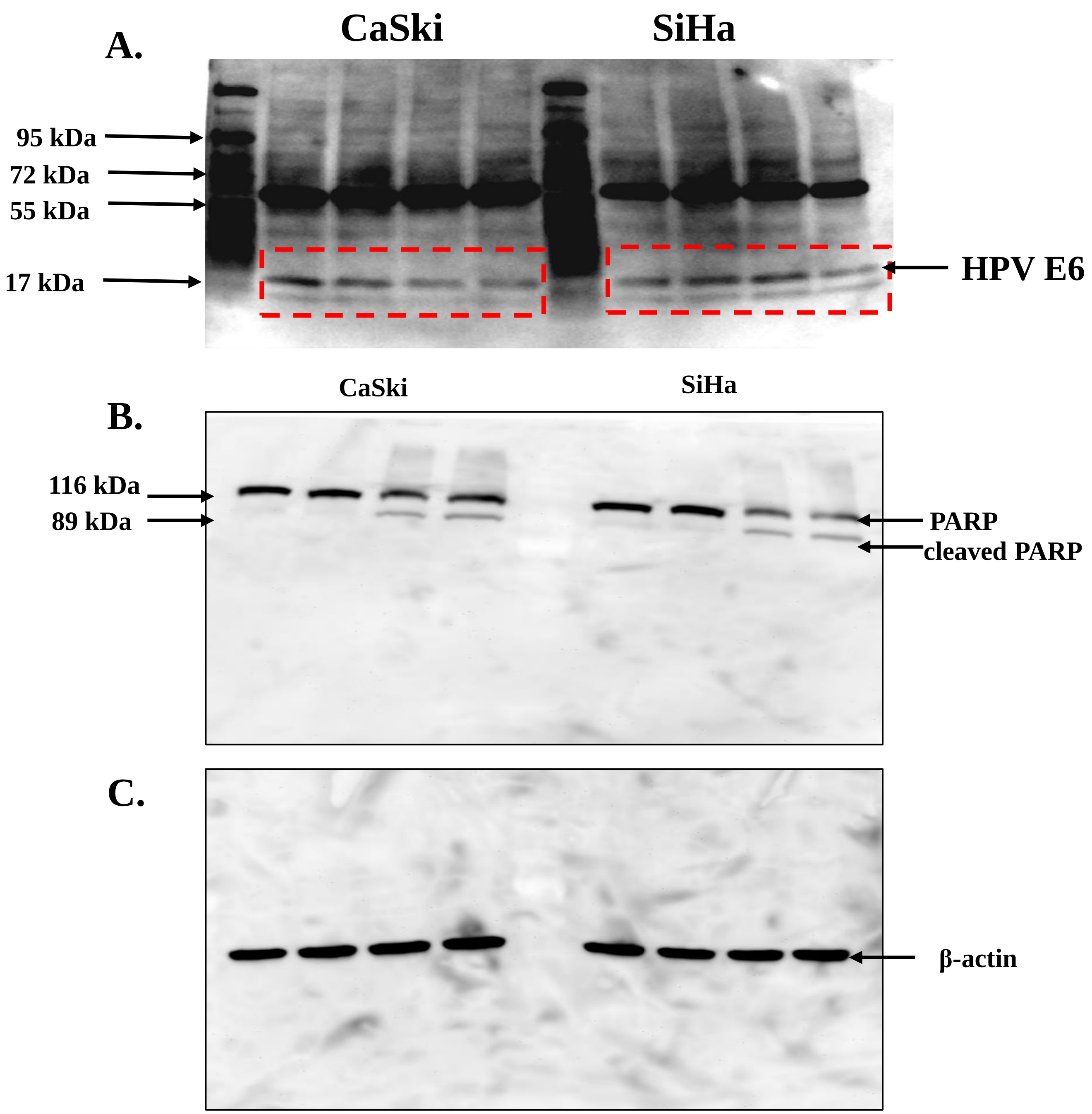

Supplementary Figure 9: A. Full blot of HPVE6 as shown in Fig. 5 Ai-ii. **B.** Full blot of PARP and to detect PARP and cleaved PARP proteins as shown in Fig. **3C i-ii. C.** β-actin for PARP as shown in 3Ci-ii..
